# 

**Published:** 1892-05-21

**Authors:** 


**Notices of Births, Deaths, and Marriages,** are inserted in this column at a charge of 2s. 6d. for an announcement not exceeding four lines.

### NOTICE TO CORRESPONDENTS.

All MS., Letters, Books for Review, and other matters intended for the Editor should be addressed The Editor, The Lodge, Porchester Square, London, W.

The Editor cannot undertake to return rejected M.S., even when accompanied by stamped directed envelope.

**Notice to Advertisers and Subscribers.**

All Advertisements, Orders for Copies of The Hospital, and Business Communications should be addressed to The Publisher, *not to the Editor*, The Hospital, 140, Strand, W.C.

The Cost of Subscription, post free, is as follows:—Per week, 2½d.; per quarter, 2s. 6d.; per half-year, 5s.; per annum, 8s. 6d. Foreign:—Per annum, 10s. 8d.; payable, in all cases, in advance.

**"Burdett's Hospital Annual," 1891—1892.**

Third year of publication. 600 pp. Boards, gilt lettering. A most complete guide to British and Colonial Hospitals, Dispensaries, Nursing and Convalescent Institutions, and Asylums. Price 3s. 6d. Published by The Scientific Press (Limited), 140, Strand, W.C.

**NOTICE.—The Index for Vol. X. (ending September, 1891), is on sale at the Office of this Journal, price 2½d., post free.**

### SCRAPS AND GLEANINGS.

THE twentieth annual collection of the Metropolitan Hospital Sunday Fund will take place on June 19th.

IT is proposed to erect a cottage hospital for Falmouth, if the money to carry on such an institution can be raised.

WE are glad to learn that the memorial to Cardinal Manning is to take the form of a refuge for the London poor, not a statue.

THE Jewish Convalescent Home (Norwood and Brighton) has received from Baron de Hirsch, through Mrs. Bischoffsheim, the sum of four hundred pounds.

THE Duke of Connaught has consented to open the new pleasure pier at Southampton, constructed by the local Harbour Board at a cost of £50,000, on June 2nd.

THE sum of £12 3s. 0½d. was collected on Hospital Sunday at the gates of the Loughinisland Catholic Church for the Mater Infirmorum Hospital in Loughinisland.

AT the general quarterly meeting of the Devon and Exeter Hospital a donation of £100 from Mr. Short, of Bickham, and another of £100 from an anonymous donor were announced.

THE Committee of the Wimbledon Cottage Hospital, in their report, announce with regret the resignation of Mr. J. F. Schwann, who has held the post of Honorary Secretary since 1876.

THE Duke of Cambridge has promised to attend a meeting at the Mansion House, on the 20th inst., for the purpose of raising funds to rebuild the Royal Ophthalmic Hospital, Moorfields.

MADAME ALBANI has kindly promised to sing at the morning concert to be given at St. James's Hall in June, in aid of the funds of the National Society for the Prevention of Cruelty to Children.

THE Queen has contributed £50 to the Royal Maternity Charity, 31, Finsbury Square, which was instituted in 1757. The triennial festival is to be held on the 17th inst.

MR. CORBETT has presented the Hill Estate, Stourbridge, comprising thirty acres and a mansion house, with money for alterations and endowments, amounting in all to £10,500, for a hospital for the district.

THE weekly return of the Registrar-General up to May 7th shows that the death-rate in London was 19·8 per thousand. There was one death from small-pox out of 16 fresh cases; and 11 deaths from influenza.

MRS. BLANCHE TURNER, of Fornham St. Martin, near Bury St. Edmunds, last week attained her 103rd birthday. Among those who called to congratulate her on the event were Earl Cadogan and Ladies Emily and Sophie Cadogan.

THE International Hospital, Naples, has just suffered a great loss in the death of its new Matron, Miss Edith Green, who had only entered on her duties scarcely two months ago, and has been carried off after a short illness.

THE late Mr. John Silvani, of Brighton, left a legacy of £10,000 to the Sussex County Hospital, with a residue of his estate estimated to reach the sum of another £20,000. Mr. Silvani also left £1,000 each to several Brighton charities.

THE Duke of Westminster has forwarded £400 to the Treasurer of the London Temperance Hospital, Hampstead Road, for the benefit of the Grosvenor Children's Ward, opened on the 11th inst. by the Duchess of Westminster. His Grace has also signified his intention of raising his annual subscription to £100.

THE *British Medical Journal* announces with great regret that Mr. W. Morrant Baker has been compelled to resign his appointment of surgeon to St. Bartholomew's Hospital on account of ill-health. The first of the assistant-surgeons, Mr. Butlin, will now become surgeon. As Mr. Baker did not lecture, no chair will be vacant.

THE annual report of the Epsom and Ewell Cottage Hospital states that the general appreciation of the work of the hospital is shown by the collection of £110 made by the committee of Ashtead, Epsom and Ewell, on Hospital Saturday. The total income for the past year was £990 13s. 10d. Against this payments amounted to £994 17s. 4d.

A PRIVATE subscription dance was held at the Addison Hall, Kensington, on Thursday, May 16th, in aid of the Metropolitan Hospital, Kingsland Road. Owing to lack of funds only 78 beds out of 160 are available for in-patients. The hospital is situated in the centre of the poorest neighbourhood in the north-east of London, with a population of 260,000.

THE Clarence Memorial Fund is being raised by St. Mary's Hospital, under the special patronage of the Queen and the Prince and Princess of Wales, to build a new wing, which is to be called the "Clarence Memorial Wing." The Queen has headed the subscription list with a donation of £100, and Prince George of Wales has become President of the hospital.

SOME two years ago the late Miss Eliza Vicary bequeathed the sum of £400 to build a women's ward to the Warminster Cottage Hospital, and Miss Maria Vicary a little later supplementing this amount by a donation of £200, the trustees at once decided to erect a wing to the hospital. This work has now been completed, and was formally opened a short time ago.

MRS. ARMITAGE, of Altricham, Manchester, has just opened a convalescent home at Clifton under the name of "The Oldland Rest Home." Accommodation has been provided for six female convalescents, and four free beds have been placed at the disposal of patients from the infirmary. These patients will remain three weeks. The other two beds are for patients from other sources, who will be charged the nominal sum of 5s. for the 21 days' visit.

THE Universal Food and Cookery Association held their sixth annual exhibition at the Portman Rooms, Baker Street, last week. During the last five years the Association made a profit of nearly £1,300, which has been divided among the various London hospitals, &c., the chief participators being the French Hospital £550; Charing Cross Hospital, £400; and the Great Northern Hospital, £1[illegible]. The exhibits were numerous and interesting, and lectures were delivered at intervals by Dr. Allinson, Mrs. Wilson, Dr. C. Charles, and others. Cookery competitions for children and for cooks of the 1st Battalions of the Grenadier and Coldstream Guards were awarded during the week.

### THE STUDENT OF MEDICINE.

[All communications for this Supplement should be addressed to The Editor of The Hospital, at 140, Strand, with the words, "Students' Column," written in the left hand top corner of the envelope.]

### APPOINTMENTS.

Anderson, W., M.B., C.M.Glas., appointed Resident Assistant Medical Officer of the Aberdeen General Infirmary. Andrew, H., M.R.C.S.Eng., L.R.C.P.Lond., appointed House-Surgeon to the Devon and Exeter Hospital. Archer, A. M., B.A., M.D., B.Ch.,Univ.Dub., L.M., appointed Medical Officer to the Chester Union Workhouse. Bowes, W. H., M.D., B.S.Lond., F.R.C.S. Eng., appointed Assistant Medical Officer to the Plymouth Borough Asylum, Ivybridge, Devon. Bushby, T., M.B., C.M. Edin., appointed Assistant Physician to the Hospital for Consumption and Diseases of the Chest, Liverpool. Cavafy, J., M.D., F.R.C.P.Lond., appointed Examiner in Medicine in the University of London. Clarke, H., M.A., M.D., M.Ch., appointed Assistant Physician to the Hospital for Consumption and Diseases of the Chest, Liverpool. Clarke, T., M.D.Q.U.I., appointed Physician to the Hospital for Consumption and Diseases of the Chest, Liverpool. Hunter, Dr., appointed Assistant Medical Officer of the Royal Asylum, Montrose. Keiffenheim-Trubridge, L. W., M.D., B.S.Durh., M.R.C.S., L.R.C.P., appointed Honorary Physician to the Chelsea, Brompton, and Belgrave Dispensary, Sloane Square, S.W. Lansdowne, R. G., M.B., B.S.Durh., M.R.C.S., appointed Anæsthetist to the Bristol General Hospital. Luff, A. P., M.D., B.Sc., M.R.C.P.Lond., appointed Examiner in Forensic Medicine in the University of London. Macintosh, D., M.B., C.M.Glas., appointed Resident Assistant Medical Officer to the Aberdeen Infirmary. Morton, C. A., L.R.C.P.Lond., M.R.C.S., appointed Registrar to the Bristol General Hospital. Pater, Dr., appointed Honorary Consulting Physician to the Sheffield Hospital. Pickels, J. A., M.R.C.S., L.R.C.P., appointed House-Surgeon to the Jessop Hospital for Women, Sheffield. Potts, W., L.R.C.P.Lond., M.R.C.S.Eng., appointed Assistant Medical Officer of the Crumpsall Workhouse, Manchester. Robertson, R., M.D., L.R.C.S.Edin., appointed Consulting Physician to the Hospital for Consumption and Diseases of the Chest, Liverpool. Watson, W. R., Kemlo, M.A., M.B., C.M. Glas., appointed Assistant Medical Officer to the Govan Poorhouse and Asylum. Wilkinson, A. T., M.D.Lond., M.R.C.P., appointed Honorary Assistant Physician to the Royal Infirmary, Manchester. Doctor John Syer Bristowe has accepted the appointment of Consulting Physician, and Mr. Christopher Heath that of Consulting Surgeon to the West End Hospital.

### VACANCIES.

#### Resident Medical Officers.

The following vacancies are announced this week, the amount of salary in each case being given after the name of the institution:

Addenbrooke's Hospital, Cambridge, Resident House-Surgeon—Salary £65 per annum, with board, &c.

Chelsea Hospital for Women, Fulham Road, S.W., Resident Medical Officer—Salary £80 per annum, with board, &c.

County Asylum, Carlisle, Junior Assistant Medical Officer—Salary £80 per annum, with board.

Clayton Hospital and Wakefield Dispensary, Wakefield, Junior House-Surgeon, unmarried—Honorarium, £25 per annum, with board, &c.

General Hospital, Nottingham, Assistant House-Surgeon. Appointment for six months. Board, &c.

Great Yarmouth Hospital, House-Surgeon; doubly qualified—Salary £90 per annum, with board, &c.

Hospital for Sick Children, Great Ormond Street, W.C., House-Physician, unmarried. Appointment for one year—Salary £60 per annum, with board, &c.

Metropolitan Asylums Board, Western Fever Hospital, Seagrave Road, Fulham, S.W., Clinical Assistant for three months. Board, &c.

Queen Charlotte's Lying-in Hospital, Marylebone Road, N.W., Resident Medical Officer. Appointment for four months—Salary at the rate of £60 per annum, with board, &c.

Royal Albert Edward Infirmary, Wigan, Junior House-Surgeon; doubly qualified—Salary £80 per annum, with apartments, &c.

#### Non-Resident Medical Officers.

General Hospital, Birmingham, Assistant Physician. Appointment for three years. Honorarium, £100 per annum.

Grosvenor Hospital for Women and Children, Vincent Square, S.W. Chloroformist.

Hospital for Sick Children, Great Ormond Street, W.C., Assistant House-Surgeon. Appointment for one year — Salary £50 per annum.

Victoria Hospital for Children, Queen's Road, Chelsea, S.W. Dental Surgeon.

The Hospital Advertiser.

